# Comparison of ESC and ACC/AHA guidelines for myocardial revascularization

**DOI:** 10.1007/s12350-017-0811-5

**Published:** 2017-02-21

**Authors:** Jim Stirrup, Alejandro Velasco, Fadi G. Hage, Eliana Reyes

**Affiliations:** 1 Nuclear Medicine Department, Royal Brompton and Harefield NHS Foundation Trust, Sydney Street, London, SW3 6NP United Kingdom; 2 Division of Cardiovascular Disease, Department of Medicine, The University of Alabama at Birmingham, Birmingham, Alabama USA; 3 Section of Cardiology, Birmingham Veteran Affairs Medical Center, Birmingham, Alabama USA; 4 King’s College London, London, United Kingdom

**Keywords:** Guidelines, imaging, revascularization, myocardial

## Abstract

In 2014, the Task Force on Myocardial Revascularization of the European Society of Cardiology and the European Association for Cardio-Thoracic Surgery with the special contribution of the European Association of Percutaneous Cardiovascular Interventions published a comprehensive set of recommendations on myocardial revascularization in patients presenting with acute or chronic coronary artery disease. In the United States, pertinent guidance on this topic has been published by the American College of Cardiology, American Heart Association and other relevant societies in multiple guideline documents that have been published in recent years. This document brings together European and American recommendations on myocardial revascularization with a focus on the role of cardiac imaging

In 2014, the Task Force on Myocardial Revascularization of the European Society of Cardiology (ESC) and the European Association for Cardio-Thoracic Surgery (EACTS) with the special contribution of the European Association of Percutaneous Cardiovascular Interventions (EAPCI) published a comprehensive set of recommendations on myocardial revascularization in patients presenting with acute or chronic coronary artery disease (CAD).^1^ In the United States, pertinent guidance on this topic has been published by the American College of Cardiology (ACC), American Heart Association (AHA), and other relevant societies in multiple guideline documents that have been published in recent years.^2^–^10^ This document brings together European and American recommendations on myocardial revascularization for side-by-side comparison; class (I, II or III) and level of evidence (A, B or C) are shown for each recommendation (Tables 1, 2, 3, 4, 5, 6 and Figures 1, 2). This is followed by two Editorial comments that reflect on the similarities and the differences between European and American guidance and the relevance of these to clinical practice. This represents the second of a new series of comparative guidelines review; the first of these focused on the recently published ACC/AHA and ESC/ESA guidelines for the cardiovascular evaluation and management of patients undergoing non-cardiac surgery.^11^–^13^

**Table 1:** Indications for diagnostic imaging in patients with suspected CAD

Recommendation	ESC/EACTS		ACC/AHA	
	Class	LOE	Class	LOE
Functional imaging^*^ is recommended in patients with intermediate^†^ probability of CAD^1^,^2^	I	A	I^‡^	B
Invasive angiography is recommended in patients with ESC: high probability of CAD^1^ ACC/AHA: unacceptable ischemic symptoms despite optimal medical therapy and who are amenable to, and candidates for, coronary revascularization^3^	I	A	I	C
CTA is recommended in patients with intermediate probability of disease^1^,^2^	IIa	A	II^§^	B
Combined or hybrid imaging^II^ is recommended in patients with intermediate probability of CAD^1^	IIa	B	NSER	
Invasive angiography is reasonable to define the extent and severity of CAD in patients with suspected SIHD whose clinical characteristics and non-invasive testing (exclusive of stress testing) results indicate a high likelihood of severe IHD and who are amenable to, and candidates for, coronary revascularization^3^	NSER		IIa	C
Invasive angiography is reasonable in patients with suspected symptomatic SIHD who cannot undergo diagnostic stress testing, or have indeterminate or non-diagnostic stress tests, when there is a high likelihood that the findings will result in important changes to therapy^3^	NSER		IIa	C
Invasive angiography is recommended in patients with intermediate probability of CAD^1^	IIb	A	NSER	
Invasive angiography might be considered in patients with stress test results of acceptable quality that do not suggest the presence of CAD when clinical suspicion of CAD remains high and there is a high likelihood that the findings will result in important changes to therapy^3^	NSER		IIb	C
Diagnostic imaging (invasive or non-invasive) is not recommended in asymptomatic patients^1^,^4^	III	A-C^¶^	III^**^	C
Diagnostic imaging (invasive or non-invasive) is not recommended in patients with low probability of CAD^1^,^2^	III	A, C^††^	II^$^	B, C
			III	C
CTA is not recommended in patients with high probability of CAD^1^	III	B	NSER	
Functional imaging is not recommended in patients with high probability of CAD^1^	III	A, B^‡‡^	NSER	
Combined or hybrid imaging is not recommended in patients with high probability of CAD^1^	III	B	NSER	

^*^Functional imaging refers to stress echocardiography, MPS, MRI, and PET imaging^1^

^†^Probability of significant CAD: Low <15%; intermediate 15-85%; high >85%^1^

^‡^ACC/AHA guidelines stipulate intermediate to high probability of CAD in this circumstance^2^

^§^This is a class IIb recommendation for patients able to exercise and a IIa for patients unable to exercise^2^

^‖^Hybrid imaging refers to systems in which two imaging modalities are combined in the same scanner (e.g., multidetector CT and SPECT, multidetector CT and PET)

^¶^LOE A for invasive angiography, stress echocardiography, and MPS; LOE B for CTA, stress MRI, and PET; LOE C for combined or hybrid imaging

^**^Per ACC/AHA guidelines, MPS may be considered in asymptomatic adults with diabetes or a strong family history of CAD, or when previous risk assessment testing suggests high risk of CAD (class IIb, LOE C)^4^

^††^LOE A for invasive angiography, stress echocardiography, and MPS; LOE C for CTA, stress MRI, PET, and combined or hybrid imaging

^$^ACC/AHA guidelines state that, in patients with low probability of CAD who are incapable of at least moderate physical exertion, CTA is a class IIa, LOE B. In patients who require testing, exercise or pharmacologic echocardiography is class II, LOE C. Exercise MPS and pharmacologic stress with MPS, echocardiography, or MRI are class III in patients with an interpretable ECG who are capable of at least moderate physical exertion

^‡‡^LOE A for stress echocardiography and MPS; LOE B for stress MRI, PET, and combined or hybrid imaging

**Table 2:** Indications for revascularization in patients with stable angina or silent ischemia according to the extent of CAD

Recommendation	ESC/EACTS		ACC/AHA	
	Class	LOE	Class	LOE
For symptoms, revascularization is recommended for				
Any significant coronary stenosis^*^ in the presence of limiting angina or angina equivalent that does not respond to medical therapy^1^,^5^	I	A	I	A
For prognosis, revascularization is recommended for				
Significant left main stenosis^1^,^5^	I	A	I	B
Any significant proximal LAD stenosis^1^,^5^	I	A	I^†^	B
Survivors of sudden cardiac death with presumed ischemia-mediated ventricular tachycardia caused by significant stenosis in a major coronary artery^5^	NSER		I	C
Two-vessel or three-vessel CAD with significant stenosis and impaired LV function^‡^ 1,5	I	A	II	B
Severe or extensive ischemia^§^ 1,5	I	B	IIa	B
Single remaining patent coronary artery with significant stenosis^1^	I	C	NSER	
Extensive anterior wall ischemia on non-invasive testing and previous CABG^5^	NSER		IIb	B
Significant stenoses in two major coronary arteries not involving the proximal LAD and without extensive ischemia^5^,^6^	NSER		IIb	C
Revascularization is not recommended in patients with one or more coronary stenoses that are not functionally or anatomically significant, involve only the left circumflex or right coronary artery, or subtend only a small area of viable myocardium^5^	NSER		III	B

^*^Defined in the ESC guidelines as coronary diameter stenosis >50% with documented ischemia on imaging, or FFR ≤0.80 for diameter stenosis <90%;^1^ and in the ACC/AHA guidelines as ≥50% left main or ≥70% non-left main or FFR ≤0.80 stenosis^5^

^†^This indication is ACC/AHA class I in the context of multivessel CAD, and class II in single-vessel disease

^‡^LVEF <40% (ESC guidelines)^1^. This indication is ACC/AHA class IIa in patients with mild-moderate LV dysfunction (LVEF, 35-50%) and class IIb in patients with severe LV dysfunction (LVEF, <35%) without significant left main CAD^5^

^§^Defined as >10% ischemic LV myocardium (ESC guidelines)^1^, or >20% perfusion defect on stress MPS, high-risk criteria on stress testing or abnormal intracoronary hemodynamic evaluation (ACC/AHA guidelines)^5^

**Table 3:** Recommendations for non-invasive evaluation before revascularization in patients presenting with an acute coronary syndrome

Recommendation	ESC/EACTS		ACC/AHA	
	Class	LOE	Class	LOE
Non-invasive documentation of inducible ischemia in low-risk NSTE-ACS patients without recurrent symptoms is recommended before deciding on invasive evaluation^1^,^7^	I	A	I	B
Non-invasive testing for ischemia should be performed before discharge in patients with STEMI who have not had coronary angiography and do not have high-risk clinical features for which coronary angiography would be warranted^8^	NSER		I	B
In initially stabilized patients, an ischemia-guided strategy may be considered for patients with NSTE-ACS (without serious comorbidities or contraindication to this approach) who have an elevated risk for clinical events^7^	NSER		IIb	B
PCI of a totally occluded infarct artery >24 hours after STEMI should not be performed in asymptomatic patients with one- or two-vessel CAD if patients are haemodynamically and electrically stable and do not have evidence of severe ischemia^8^	NSER^*^		III	B

^*^According to ESC guidance, “in patients presenting days after an acute event, only those with recurrent angina or documented residual ischemia and proven viability on non-invasive imaging in a large myocardial territory may be considered for revascularization when the infarct artery is occluded”^1^

**Table 4:** Recommendations on revascularization in patients with chronic heart failure and systolic LV dysfunction according to the presence of viable and /or scarred myocardium

Recommendation	ESC/EACTS		ACC/AHA	
	Class	LOE	Class	LOE
Myocardial revascularization should be considered in the presence of viable myocardium^*^ 1,5,9	IIa	B	IIa^†^	B
CABG with surgical ventricular restoration may be considered in patients with scarred LAD territory^‡^ 1,9	IIb	B	IIb	B
PCI may be considered if anatomy is suitable, in the presence of viable myocardium, and surgery is not indicated^1^	IIb	C	NSER	
CABG might be considered with the primary or sole intent of improving survival in patients with SIHD and severe LV systolic dysfunction (EF, <35%) whether or not viable myocardium is present^5^,^6^	NSER^§^		IIb	B

^*^According to ESC guidelines, “nuclear imaging techniques have a high sensitivity for the detection of viability whereas techniques evaluating contractile reserve have lower sensitivity but higher specificity. Differences in performance between the various techniques are small; experience and availability often determine which technique is used”^1^

^†^CABG is recommended to improve survival in patients with a) target vessels supplying a large area of viable myocardium; b) mild to moderate LV systolic dysfunction (LVEF, 35-50%) and significant multivessel CAD or proximal LAD stenosis when viable myocardium is present in the region of intended revascularization^5^,^6^

^‡^“Especially if a post-operative LV end-systolic volume index <70mL/m^2^ can be predictably achieved” 1. ACC/AHA guidelines discuss surgical reverse remodeling or LV aneurysmectomy in isolation, with a IIb recommendation in carefully selected patients with HFrEF for specific indications, including intractable heart failure and ventricular arrhythmias^9^

^§^ESC guidelines recommend CABG to improve prognosis in patients with severe LV dysfunction and significant LAD stenosis and multivessel CAD but do not specify the state of viability (class I, LOE B)^1^

**Table 5:** Recommendations for stress testing and ischemia-guided revascularization in special groups

Recommendation	ESC/EACTS		ACC/AHA	
	Class	LOE	Class	LOE
In stable patients with diabetes, multivessel CAD, and/or evidence of myocardial ischemia, revascularization is indicated to reduce cardiac adverse events^1^,^5^	I	B	IIa^*^	B
Repeat revascularization is indicated in post-CABG patients with severe symptoms or extensive ischemia despite medical therapy if technically feasible^1^,^5^	I	B	II^†^	C
Stress testing should be considered in patients with a primary indication for CABG and moderate mitral valve regurgitation to determine the extent of ischemia and regurgitation^1^	IIa	C	NSER	
In patients with CAD and LVEF <35%, testing for residual ischemia and subsequent revascularization should be considered prior to primary prophylactic ICD implantation^1^	IIa	B	NSER	
Prophylactic myocardial revascularization before high-risk vascular surgery may be considered in stable patients if they have persistent signs of extensive ischemia or are at high cardiac risk^‡^	IIb	B	NSER^§^	

^*^This indication refers to the preference of CABG over PCI in patients with diabetes and multivessel disease, particularly if a LIMA graft can be anastomosed to the LAD artery^5^,^6^

^†^This is a class IIa indication for PCI and class IIb for repeat CABG^5^,^6^

^‡^High cardiac risk (reported cardiac risk >5%): (1) aortic and other major vascular surgery; (2) peripheral vascular surgery^1^

^§^Revascularization before non-cardiac surgery is recommended when indicated by existing clinical practice guidelines^10^

**Table 6:** Strategies for follow-up and management after myocardial revascularization

Recommendation	ESC/EACTS		ACC/AHA	
	Class	LOE	Class	LOE
Asymptomatic patients				
Early stress testing with imaging should be considered in specific patient subsets^*^	IIa	C	NSER	
Routine stress testing may be considered >2 years after PCI and >5 years after CABG^1^	IIb	C	IIa^†^	C
Standard exercise ECG performed ≥1-year intervals might be considered in patients with prior evidence of silent ischemia, or at high risk for a recurrent cardiac event who can exercise and have an interpretable ECG^2^	NSER		IIb	C
Control angiography (CTA or invasive) within 3-12 months of high-risk PCI (e.g., unprotected left main stenosis) may be considered, irrespective of symptoms^1^	IIb	C	NSER	
Symptomatic patients				
Stress testing is recommended in patients with new or worsening symptoms not consistent with unstable angina^‡^ 2,11	I	C	I	B
It is recommended to reinforce medical therapy and lifestyle changes in patients with low-risk findings (e.g., <5% ischemic myocardium) on stress testing^1^,^11^	I	C	NSER	
Coronary angiography is recommended in patients with intermediate-to-high-risk findings^§^ on stress testing^1^	I	C	NSER	
CTA for assessment of patency of CABG or of coronary stents ≥3 mm in diameter might be reasonable in patients with new or worsening symptoms not consistent with unstable angina irrespective of ability to exercise^2^	NSER		IIb	B
CTA might be reasonable in patients with new or worsening symptoms not consistent with unstable angina in the absence of known moderate or severe calcification or to assess patency of coronary stents <3 mm in diameter, irrespective of ability to exercise^2^	NSER		IIb	B
CTA is not recommended for the assessment of native coronary arteries with known moderate or severe calcification or with coronary stents <3 mm in diameter in patients with new or worsening symptoms not consistent with unstable angina, irrespective of ability to exercise^2^	NSER		III	B

^*^This includes the following: High-safety professions (e.g., pilots, drivers, divers), competitive athletes, patients engaging in strenuous recreational activities, sudden death survivors, patients with diabetes—especially if insulin-requiring, patients with incomplete or suboptimal revascularization, complicated course during revascularization, or multivessel CAD and residual intermediate lesions or with silent ischemia^1^

^†^This recommendation is specific to the assessment of patients with prior evidence of silent ischemia or who are at high risk for a recurrent cardiac event and (a) are unable to exercise adequately, or (b) have an uninterpretable ECG, or (c) have a history of incomplete coronary revascularization^2^

^‡^According to ESC guidelines, stress imaging (stress MPS, echocardiography or MRI) is preferred over the exercise ECG^14^. ACC/AHA guidelines recommend standard exercise ECG in patients who are able to exercise and have an interpretable ECG. Stress imaging is indicated in patients with an uninterpretable ECG and in those unable to exercise adequately. Stress imaging is also reasonable in patients who (a) previously required imaging with exercise stress, or (b) have known multivessel CAD, or (c) have a high risk for multivessel CAD (class IIa, LOE B)^2^

^§^Ischemia at low workload, early onset ischemia, multiple areas of high-grade wall motion abnormality, or reversible perfusion defect^1^

**Figure 1:**
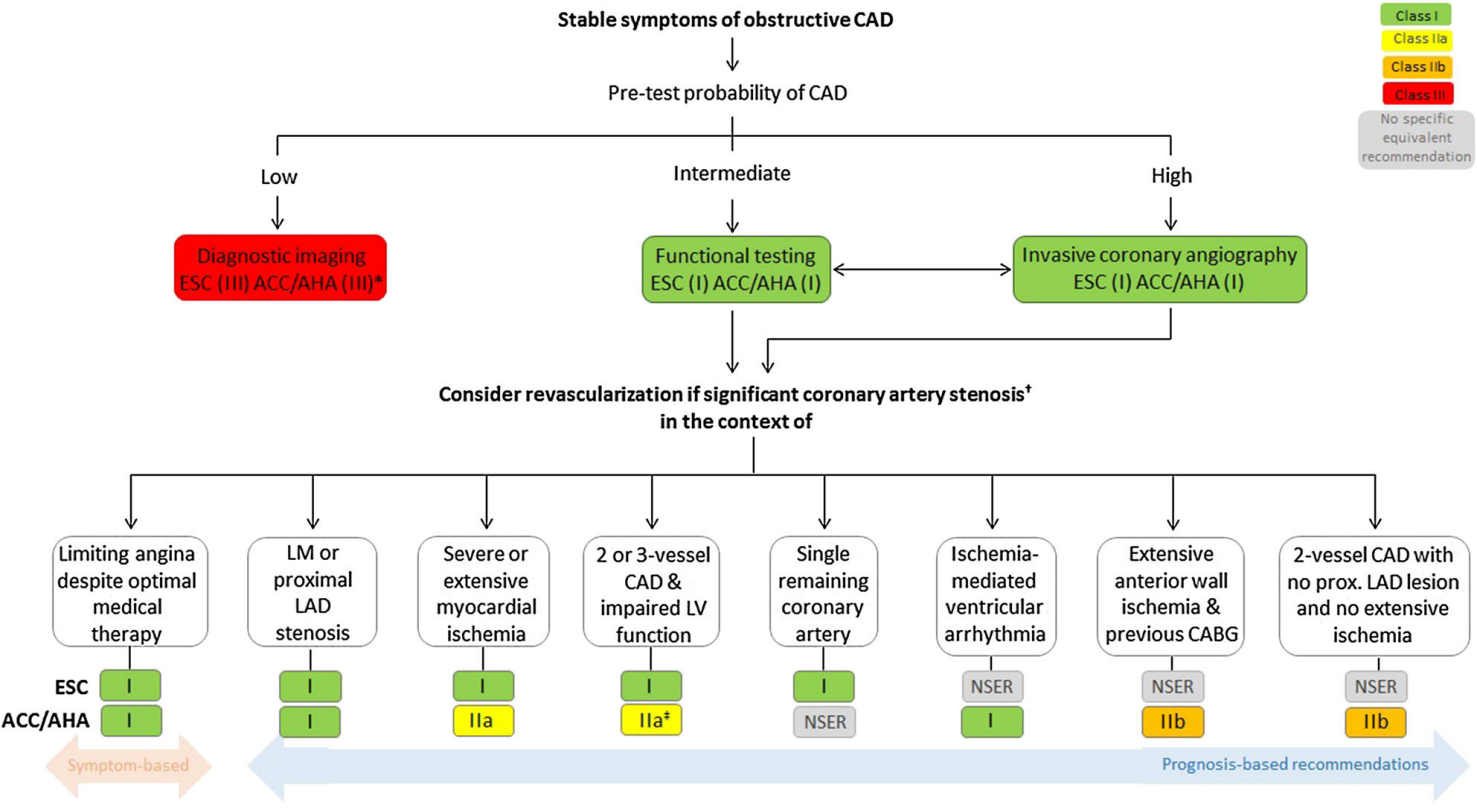
Indications for coronary revascularization in patients with suspected obstructive CAD per ESC/EACTS and ACC/AHA guidelines. ^*^CTA and stress echocardiography are ACC/AHA class II indication. ^†^Defined as >50% coronary diameter stenosis with documented ischaemia on non-invasive imaging, or FFR ≤ 0.80 for diameter stenosis <90% (ESC guidelines); ≥50% left main, or ≥70% non-left main, or FFR ≤0.80 stenosis (ACC/AHA guidelines). ^‡^This is a class IIb indication in patients with LVEF <35%. *CABG*, coronary artery bypass grafting; *CAD*, coronary artery disease; *LAD*, left anterior descending; *LM*, left main

**Figure 2:**
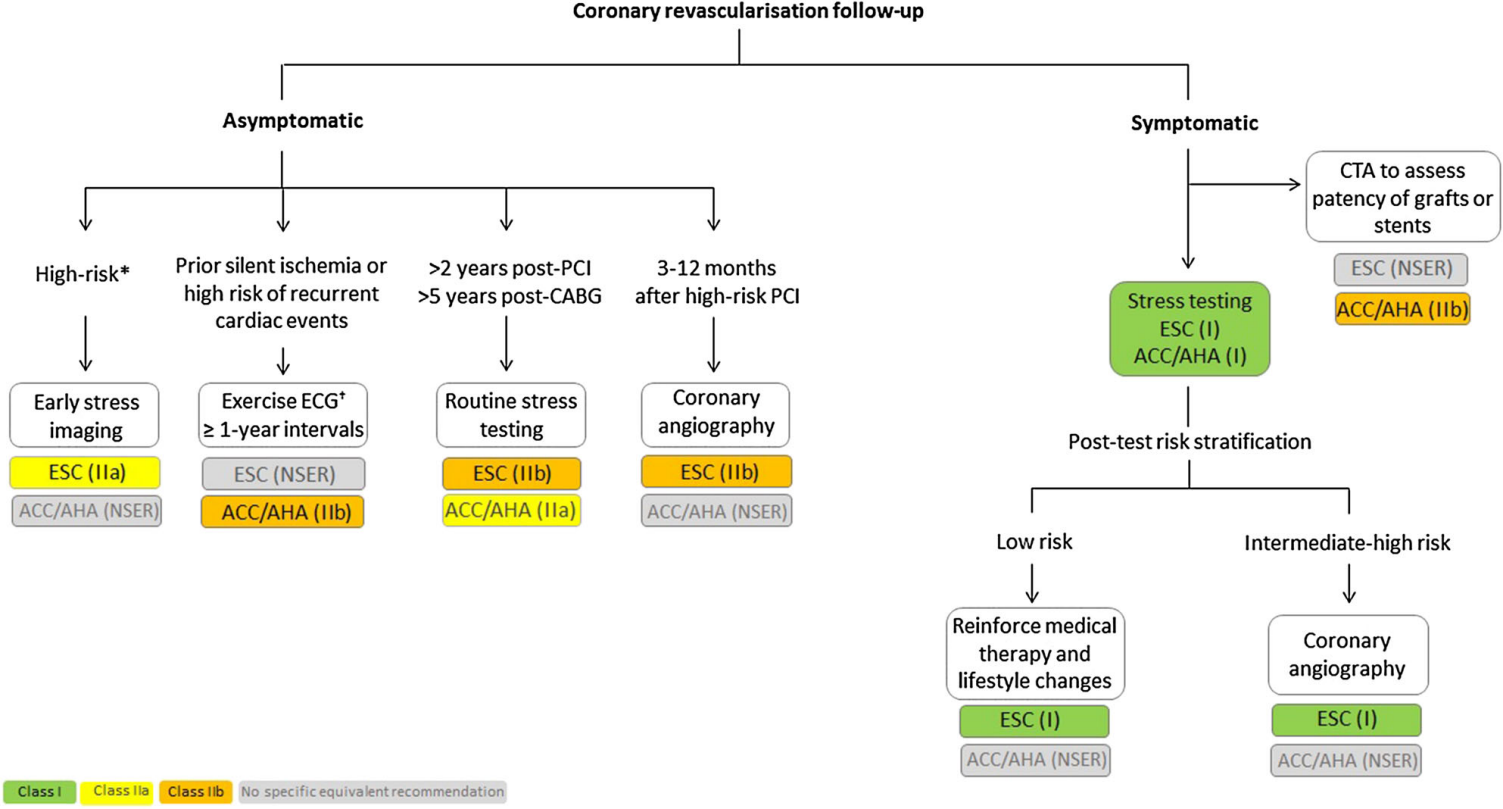
ESC/EACTS and ACC/AHA guidance for the assessment of patients after coronary revascularization according to the presence of symptoms. ^*^This includes the following: High-safety professions (e.g., pilots, drivers, divers), competitive athletes, patients engaging in strenuous recreational activities, sudden death survivors, patients with diabetes—especially if insulin-requiring, patients with incomplete or suboptimal revascularization, complicated course during revascularization, or multivessel CAD and residual intermediate lesions or with silent ischemia. ^†^This recommendation is most appropriate in patients who can exercise adequately and have an interpretable ECG. *CABG*, coronary artery bypass grafting; *CTA*, computed tomographic angiography; *PCI*, percutaneous coronary intervention

## Abbreviations

CABG: Coronary artery bypass grafting
CAD: Coronary artery disease
CTA: Computed tomographic angiography
EF: Ejection fraction
FFR: Fractional flow reserve
HFrEF: Heart failure with reduced ejection fraction
ICD: Implantable cardioverter defibrillator
LAD: Left anterior descending coronary artery
LIMA: Left internal mammary artery
LOE: Level of evidence
LV: Left ventricular
MPS: Myocardial perfusion scintigraphy
MRI: Magnetic resonance imaging
NSER: No specific equivalent recommendation
NSTE-ACS: Non-ST-segment elevation acute coronary syndrome
PCI: Percutaneous coronary intervention
PET: Positron emission tomography
SIHD: Stable ischaemic heart disease
SPECT: Single-photon emission computed tomography
STEMI: ST-segment elevation myocardial infarction
